# Assessment of the Effectiveness of Patient Education and Interviews in Improving Medication Adherence of Renal Transplant Recipients

**DOI:** 10.7759/cureus.33010

**Published:** 2022-12-27

**Authors:** Naile Akşit, Ayfer Özbaş, Serkan Akıncı

**Affiliations:** 1 Health Sciences, Fenerbahçe University, Istanbul, TUR; 2 Nursing, Istanbul University, Istanbul, TUR; 3 Health Sciences, Istanbul Gelisim University, Istanbul, TUR

**Keywords:** nonadherence, ımmunosuppressive agent, treatment efficacy, renal transplantation, drug adherence

## Abstract

Background

Non-compliance with immunosuppressive drugs has been reported as the most significant cause of graft loss. Since non-compliance with immunosuppressive drugs is preventable, certain approaches based on the risk factors and causes of non-compliance can help eliminate this problem.

Aims

The purpose of this study is to assess the effectiveness of patient education and interviews in improving medication adherence of renal-transplant recipients.

Materials and methods

This study was designed as a randomized controlled trial. Using the G*Power program, the sample size was calculated as 60 subjects, with 30 in both the intervention group and control group. Data collection tools included a patient information form, a pretest-posttest, a drug monitoring form for kidney transplant patients, the Immunosuppressive Therapy Adherence Scale, measurement of tacrolimus blood levels, and a training booklet.

Results

The mean knowledge score in the intervention group was 12.17±3.39 at baseline, and it increased to 20.73±1.57 after the intervention. The mean scores on the Immunosuppressant Therapy Adherence Scale were 11.67±0.55 and 10.70±0.99 in the intervention group and control group, respectively. There was a statistically significant difference between the pre-test and post-test means on the Immunosuppressant Therapy Adherence Scale in the intervention group. The mean Immunosuppressant Therapy Adherence Scale score was higher in the intervention group. In the measurement of tacrolimus blood levels, medication adherence was found the be higher in the intervention group. The difference between the groups was statistically significant. There was a positive correlation between the tacrolimus blood levels and the Immunosuppressant Therapy Adherence Scale scores in both groups.

Conclusions

To conclude, our results have demonstrated that patient education and interviews improve immunosuppressant medication adherence in renal transplant recipients. Using direct or indirect methods proved similar outcomes, suggesting that both evaluation methods are safe.

## Introduction

Kidney transplantation has recently become a treatment choice for patients with renal failure. The recipient's rejection of the organ (graft) is one of transplant patients' most critical issues. Non-compliance with immunosuppressive drugs has been reported as the most significant cause of graft loss [1,2]. Nearly 60% of all late acute rejections and 30-35% of graft losses are caused by non-compliance. Also, re-hospitalization due to drug non-compliance and expensive anti-rejection drugs for preventing rejection has increased treatment costs [3,4]. Prevention of graft loss is achieved by maintaining a balance between effectiveness and applicability in immunosuppressive drugs [5-7]. The World Health Organization (WHO) defines drug compliance as "the extent to which an individual's behavior adheres to the accepted recommendations of a healthcare institution". Small deviations in the dosage and timing of drug administration can be enough to boost the risk of negative outcomes [8]. Blaseer et al. (2009) found that non-compliance with immunosuppressive drugs has detrimental economic effects and short- and long-term clinical outcomes [9].

Compliance with immunosuppressive drug regimens is key for preventing graft loss and morbidity in transplant patients, but a significant portion of kidney recipients have low compliance with immunosuppressive drugs [10,11]. In kidney transplants, outcomes are significantly affected by the recipient's ability to comply with a complex and ongoing self-management regime [3,12]. Since non-compliance with immunosuppressive drugs is preventable, certain approaches based on the risk factors and causes of non-compliance can help eliminate this problem [13,14]. 

For immunosuppressive drugs whose therapeutic responses cannot be accurately measured, evaluating what concentration they reach in the blood is crucial to know the effectiveness of treatment. Several approaches are used to determine drug enforcement in individuals undergoing solid organ transplantation. Direct methods involve monitoring drug levels in blood and urine. In contrast, in indirect methods, patient outcomes are evaluated by interviews with patients or relatives, prescription records, patient diaries, questionnaires, and electronic monitoring. The literature reports various advantages for either method. Indirect methods are easy to perform and analyze, cost-effective, and give detailed information on compliance factors. Direct methods are more objective, so they have higher reliability. There is a lack of studies regarding which method is safer [15,16]. 

The transplant team must identify and solve these problems using practices to increase drug compliance after kidney transplantation. However, nurses have major responsibilities due to their close relationships with patients. Besides, the WHO states that nurses can be very effective in drug compliance by increasing patients' perceptions regarding drug compliance, developing methods to increase and evaluate compliance, and revealing the causes of non-compliance [8]. It remains unclear what needs to be done to develop and implement effective approaches to increase compliance with immunosuppressive drugs. Research suggests that behavioral approaches or a combination of behavioral, educational, and effective approaches may be more effective at increasing compliance [17,18]. Thus, as a part of clinical management, these approaches need to gain momentum in practice. There is not enough research on practices to improve compliance. Therefore, a systematic review is urgently needed to evaluate the significant approaches to increasing immunosuppressive drug compliance. Based on this information, this study was planned to evaluate the effectiveness of training and interviews to increase drug compliance in kidney transplant patients.

## Materials and methods

Objective and type of research

This study was planned and carried out as a single-center experimental randomized control trial to evaluate the effectiveness of training and interviews to increase drug compliance in kidney transplant patients.

Research hypotheses

There were four main hypotheses in this study: H1) the experimental group will have higher drug compliance after training and interviews than the control group; H2) the experimental group will have a higher level of knowledge following training after kidney transplantation; H3) tacrolimus blood levels will be at a level of agreement between the experimental and control groups; and H4) tacrolimus blood level measurements, a direct method used to evaluate immunosuppressive drug compliance, and the Immunosuppressive Therapy Adherence Scale (ITAS) scale, an indirect method, will have similar outcomes.

Population and sampling

The study population consisted of all patients hospitalized in the organ transplant service of a private hospital, Istanbul Memorial Hizmet Hospital, between May 2018 and July 2019. By using the G*Power version 3.1.9.4 program to determine the sample size with statistical power analysis, it was decided to use the t-test in independent groups based on patient education and ITAS scale score variables. Accordingly, in determining the sample size according to the reference study, the alpha bidirectional type one error value was accepted as 0.05 and power as 0.80. It was determined that at least 30 people would be included in the study by calculating the standardized effect size of 1.0 [18]. Considering the losses in the research, a total of 60 people, 30 people in each group, were reached. Those who met the inclusion criteria were assigned to either group by using www.random.org. The inclusion and exclusion criteria can be seen in Table 1.

**Table 1:** Inclusion and exclusion criteria in the study

Inclusion criteria in the study	Exclusion criteria in the study
Kidney transplant and scheduled for discharge	Kidney graft non-functional at runtime
18 years or older	Patients with a previous kidney transplant
Able to use immunosuppressive drugs independently	
Having no intellectual and auditory disabilities for learning	
Those who do not have a history of psychiatric disorders and do not use drugs for this purpose	
Those who agree to participate in the research voluntarily	
Patients who have no problem in speaking and understanding Turkish	

Data collection tools

Data collection tools included a patient information form, a pretest-posttest, a drug monitoring form for kidney transplant patients, the Immunosuppressive Therapy Adherence Scale, measurement of tacrolimus blood levels, and a training booklet.

Patient information form

The form consists of two parts, the first of which has nine questions (age, sex, marital status, educational status, social security, whom they live with), and the second part of which has eight questions (donor type, the reason for kidney transplantation, continuously used immunosuppressive and other drugs, information status, and the source of this information).

Pretest-posttest

The pretest-posttest consists of 22 questions with the choices "true", "false", and "do not know". The researcher generated the questions based on the literature to assess patients' drug awareness, misunderstandings, shortcomings, and the impact of training on their level of knowledge [1,4,9,14,19]

Drug monitoring form for kidney transplant patients

The researcher created this form to determine the effects of the drugs, as well as their administration period, duration of use, and expiration date. The form has significance in terms of making sure that drug use is under the self-control of patients, enabling drug use to be monitored by both patients and healthcare providers, providing information on how and how often the drugs should be used, and preventing non-compliance by reminding against forgetting to take the drugs or overdosing.

Immunosuppressive Therapy Adherence Scale (ITAS)

The scale was developed by Chisholm et al. in 2004 to measure adherence to immunosuppressive therapy after organ transplantation in the USA (α=0,81). Bayhan conducted a validity and reliability study in Turkish in 2014. The scale was found to have a Cronbach's alpha coefficient of α=0.65 and an item-total score correlation coefficient between 0.27-0.69. ITAS was created as a four-point Likert-type scale to evaluate compliance with immunosuppressive therapy within the last three months.

Training booklet

This booklet included the drugs used after kidney transplantation, how to use them, their effects, side effects, significance, interactions, foods, conditions to consult a physician, and rejection symptoms. The researcher calculated the readability of the training booklet using the Ateşman and Simple Measure of Gobbledygook (SMOG) readability formulas. According to Ateşman, readability is scored as 66, 54, and 67 for parts of text selected from the beginning, middle, and end, respectively, considered "standard" and at "eighth to ninth grade". According to SMOG, words with three or more syllables from ten sentences each from the beginning, middle, and end were counted, reaching a total of 65, and readability was determined in 11th grade [20]. The drugs used after kidney transplantation, how they will be used, effects, side effects, importance, drugs and foods with which it interacts, conditions that should consult a physician, and symptoms of rejection are included in the booklet.

Measurement of tacrolimus blood levels

Tacrolimus blood level was measured at the end of the study (sixth month) to determine the patient's drug compliance levels. They were told to skip their morning dose and to take it after blood was taken. Normal blood tacrolimus level in the sixth month ranges between 8-12 ng/mL according to the protocol of the hospital where the study was conducted. Tacrolimus drug compliance was evaluated according to this range.

Data collection

First Phase

The training preparing to increase immunosuppressive drug adherence after kidney transplantation consisted of five stages: analysis, design, development, application, and evaluation (ADDIE). ADDIE was created in line with the instructional design principles and process [21]. The training was given to each patient in the experimental group as individual training in their rooms. The patients were informed about the research, and their written consent was obtained. Then, the pretest-posttest was applied to evaluate their level of knowledge before the training. Some patients filled out the form themselves, the researcher asked others the questions, and the researcher filled out the form. After the evaluation, immunosuppressive drug compliance training was given to the patients in the experimental group. The duration of training lasted an average of 45 minutes. The pretest-posttest was applied again to evaluate whether the training reached its purpose. The form was evaluated by the researcher and the training was repeated for patients' incorrect information. The researcher evaluated the form and repeated training for patients' incorrect information. The educational content was given to the patients as a booklet. The drug monitoring form for kidney transplant patients was given to the patients after the training, and they were taught how to use it. Patients also received routine hospital training.

Second Phase

The training was repeated by calling the patients at the first, third, and sixth months. In the sixth month, immunosuppressive drug compliance was evaluated using the drug adherence scale and the drug monitoring form. Tacrolimus blood levels were evaluated by calling the patients for control. The patients were told to skip their morning dose and to take it after the blood was taken. The control group only received routine hospital training. In the 6th month, the researcher called the patients, the ITAS was applied, and Tacrolimus blood levels were evaluated. The patients were again told to skip their morning dose and to take it after blood was taken. Telephone numbers were obtained from the participants during the research permit.

Ethical considerations

Approval (Annex-6) was obtained from the Memorial Hizmet Hospital Group on January 18th, 2018, and ethics committee approval was obtained from the Clinical Research Ethics Committee of Istanbul Medical Faculty on April 24th, 2018. The patients were informed in detail about the research before participation, and verbal and written consent was obtained.

Statistical analysis

The Number Cruncher Statistical System (NCSS) 2007 (Kaysville, Utah, USA) software was used for statistical analysis. Descriptive statistical methods were used (mean, standard deviation, median, frequency, ratio, minimum, maximum). The conformity of quantitative data to normal distribution was tested using the Kolmogorov-Smirnov and Shapiro-Wilk tests and graphical evaluations. The student's t-test was used to compare normally distributed quantitative data between two groups, and the Mann-Whitney U test was used to compare non-normally distributed data between two groups. Pearson's Chi-Squared test, Fisher-Freeman-Halton Exact test, and Fisher's Exact test were used to compare qualitative data. The level of significance was set at p<0.05.

## Results

Demographic findings

The patients in the experimental group had a mean age of 42.67±14.88, and the patients in the control group had a mean age of 44.53±12.44 years (Table 2). Most of the patients were female (experimental: 56.7%, control: 60.0%) and primary school graduates (experimental: 36.7%, control: 36.7%), and 66.7% were married. We found that most of the patients had children (experimental: 66.7%, control: 76.7%), most lived with their spouses and children (experimental: 53.3%, control: 63.3%), were unemployed (experimental: 66.7%, control: 56.7%), were housewives (experimental: 33.3%, control: 33.3%), and had health insurance (experimental: 93.3%, control: 100%). There was no statistically significant difference between the experimental and control groups regarding demographic characteristics (p>0.05).

**Table 2:** Demographic findings according to experimental and control groups ^a^Student's t-test ^b^Pearson's Chi-squared test ^c^Fisher-Freeman-Halton test ^d^Fisher's exact test

Variables		Experimental (n=30)	Control (n=30)	p-value
		n (%)	n (%)	
Age (years)	Min-max (median)	18-70 (44)	19-67 (46.5)	^a^0.600
	Mean±SD	42.67±14.88	44.53±12.44	
Sex	Female	17 (56.7)	18 (60.0)	^b^0.793
	Male	13 (43.3)	12 (40.0)	
Education status	Literate	2 (6.7)	2 (6.7)	^c^1.000
	Primary	11 (36.7)	11 (36.7)	
	Secondary/high school	10 (33.3)	10 (33.3)	
	University	7 (23.3)	7(23.3)	
Marital status	Single	10 (33.3)	10 (33.3)	^b^1.000
	Married	20 (66.7)	20 (66.7)	
Children	Yes	20 (66.7)	23 (76.7)	^b^0.390
	No	10 (33.3)	7 (23.3)	
Lives with	Alone	0 (0)	2 (6.7)	^c^0.346
	With spouse	4 (13.3)	1 (3.3)	
	With spouse and children	16 (53.3)	19 (63.3)	
	With children	1 (3.3)	2 (6.7)	
	Other	9 (30.0)	6 (20.0)	
Employment	Employed	6 (20.0)	11 (36.7)	^c^0.338
	Unemployed	20 (66.7)	17 (56.7)	
	Unemployed due to disease	4 (13.3)	2 (6.7)	
Occupation	Civil servant	1 (3.3)	3 (10.0)	^c^0.787
	Self-employed	5 (16.7)	5 (16.7)	
	Retired	6 (20.0)	5 (16.7)	
	Worker	2 (6.7)	4 (13.3)	
	Student	2 (6.7)	0 (0)	
	Housewife	10 (33.3)	10 (33.3)	
	Other	4 (13.3)	3 (10.0)	
Health insurance	Yes	28 (93.3)	30 (100)	^d^0.492
	No	2 (6.7)	0 (0)	

Clinical findings

Regarding donor type, we found that most patients had transplantation from a living donor (experimental: 96.7%, control: 83.3%), and the donors were mostly their siblings (experimental: 31%, control: 32%) (Table 3). There was no statistically significant difference between the patients in the experimental and control groups regarding the donor types (p>0.05). Considering the new drugs that the patients in the experimental group started after kidney transplantation, 93.3% used CellCept, 93.3% used Prograf, 90.0% used Bactrim, 90.0% used Deltacortril, 86.7% used Valcyte, 73.3% used Triflucan, 60.0% used Panto, and 43.3% used other drugs. Of the patients in the control group, 100% used CellCept, 100% used Prograf, 100% used Bactrim, 100% used Deltacortril, 90% used Valcyte, 73.3% used Triflucan, 66.7% used Panto, and 43.3% used other drugs. We found that most patients continuously used other drugs (experimental: 60.0%, control: 56.7%), and these were mostly antihypertensive drugs (experimental: 38.9%, control: 70.6). We found that 90.0% of the patients in the experimental group and 100% of the patients in the control group were informed about drug use. 81.5% of the patients in the experimental group received information from their nurses, and 86.7% of the patients in the control group received information from their physicians; both groups thought that the information they received was sufficient (experimental: 70.4%, control: 76.7%). The rate of receiving information from physicians was lower in the experimental group than in the control group (p=0.019; p<0.05). The rate of receiving information from nurses was higher in the experimental group than in the control group (p=0.001; p<0.01). There was no significant difference between the groups regarding clinical characteristics or other variables (p>0.05).

**Table 3:** Clinical characteristics according to experimental and control groups ^b^Pearson's Chi-squared test ^c^Fisher-Freeman-Halton test ^d^Fisher's exact test ^e^Mann-Whitney U test

Variables		Experimental (n=30)	Control (n=30)	p-value
		n (%)	n (%)	
Donor type	Living	29 (96.7)	25 (83.3)	^d^0.195
	Cadaver	1 (3.3)	5 (16.7)	
Donor (n=54)	Mother	6 (20.7)	5(20.0)	^d^0.533
	Father	0 (0)	2 (8.0)	
	Spouse	2 (6.9)	4 (16.0)	
	Sibling	9 (31.0)	8 (32.0)	
	Child	1 (3.4)	0 (0)	
	Father + relative	1 (3.4)	0 (0)	
	Relative	4 (13.8)	3 (12.0)	
	Cross transplant	5 (17.2)	1 (4.0)	
	Unknown	1 (3.4)	2 (8.0)	
New drugs after kidney transplant	Cellcept	28 (93.3)	30 (100)	^d^0.492
	Panto	18 (60.0)	20 (66.7)	^b^0.592
	Triflucan	22 (73.3)	22 (73.3)	^b^0.001**
	Prograf	28 (93.3)	30 (100)	^d^0.492
	Bactrim	27 (90.0)	30 (100)	^d^0.237
	Deltacortril	27 (90.0)	30 (100)	^d^0.237
	Valcyte	26 (86.7)	27 (90.0)	^b^0.001**
	Other	13 (43.3)	13 (43.3)	^b^0.001**
Other continuously used drugs	Yes	18 (60.0)	17 (56.7)	^b^0.793
	No	12 (40.0)	13 (43.3)	
Drugs used (n=35)	Antifungal	1 (5.6)	0 (0)	^c^0.374
	Antihypertensive	7 (38.9)	12 (70.6)	
	Antidiabetic	2 (11.1)	3 (17.6)	
	Other	2 (11.1)	1 (5.9)	
	Antihypertensive + Antidiabetic	1 (5.6)	0 (0)	
	Antihypertensive + other	4 (22.2)	1 (5.9)	
	Antifungal + Antihypertensive+other	1 (5.6)	0 (0)	
Receiving information about drug use	Yes	27 (90.0)	30 (100)	^d^0.237
	No	3 (10.0)	0 (0)	
Source of information (n=57)	Physician	16 (59.3)	26 (86.7)	^b^0.019*
	Nurse	22 (81.5)	6 (20.0)	^b^0.001**
	Organ transplant coordinator	2 (7.4)	0 (0)	^d^0.220
	People who had a kidney transplant	1 (3.7)	0 (0)	^d^0.474
Perception of information	Sufficient	19 (70.4)	23 (76.7)	^c^0.544
	Somewhat sufficient	8 (29.6)	6 (20.0)	
	Insufficient	0 (0)	1 (3.3)	

Pretest-posttest findings for the experimental group

The mean knowledge level score of the patients in the experimental group was 12.17±3.39 before the training and 20.73±1.57 after the training (Table 4). A statistically significant difference was found between the mean scores before and after the training (p=0.001; p<0.01).

**Table 4:** Comparison of the mean pretest and posttest scores of the experimental group ^f^Wilcoxon signed-rank test **p<0,01

		Before training (n=30)	After training (n=30)	p-value
Knowledge level score	Min-max (median)	7-22 (12)	16-22 (21)	^f^0.001**
	Mean±SD	12.17±3,39	20.73±1.57	

Immunosuppressive Therapy Adherence Scale findings

Total ITAS scores ranged from nine to 12, with a mean of 11.18±0.93. Mean total ITAS scores were 11.67±0.55 for the experimental group and 10.70±0.99 for the control group (Table 5). There was a statistically significant difference between total ITAS scores regarding education status (p=0.001; p<0.01). Also, total ITAS scores were higher in the experimental group than in the control group.

**Table 5:** Evaluation of the Immunosuppressive Therapy Adherence Scale scores according to experimental and control groups ^e^Mann-Whitney U rest **p<0,01 ITAS - Immunosuppressive Therapy Adherence Scale

			Experimental (n=30)	Control (n=30)	p-value
ITAS score	Min-max (median)	9-12 (11,5)	10-12 (12)	9-12 (10)	^e^0.001**
	Mean±SD	11.18±0.93	11.67±0.55	10.70±0.99	

Tacrolimus blood levels and their comparison with ITAS scores

Based on the tacrolimus measurements of the experimental group, drug compliance was higher in the experimental group than in the control group, with a statistically significant difference (p<0.05) (Table 6).

**Table 6:** Evaluation of tacrolimus levels according to experimental and control groups ^a^Student's t-test ^b^Pearson's Chi-squared test

		Total (n=60)	Experimental (n=30)	Control (n=30)	p-value
Tacrolimus levels	Min-max (median)	5.8-12.7 (8.60)	6.4-12.1 (8.8)	5.8-12.7 (8.1)	^e^0.022
	Mean±SD	8.58±1.50	8.82±1.24	8.35±1.71	
Adherence		31 (56.6)	19 (63.3)	12 (40.0)	^b^0.031
Non-adherence		29 (44.4)	11 (36.7)	18 (60.0)	

**Figure 1:**
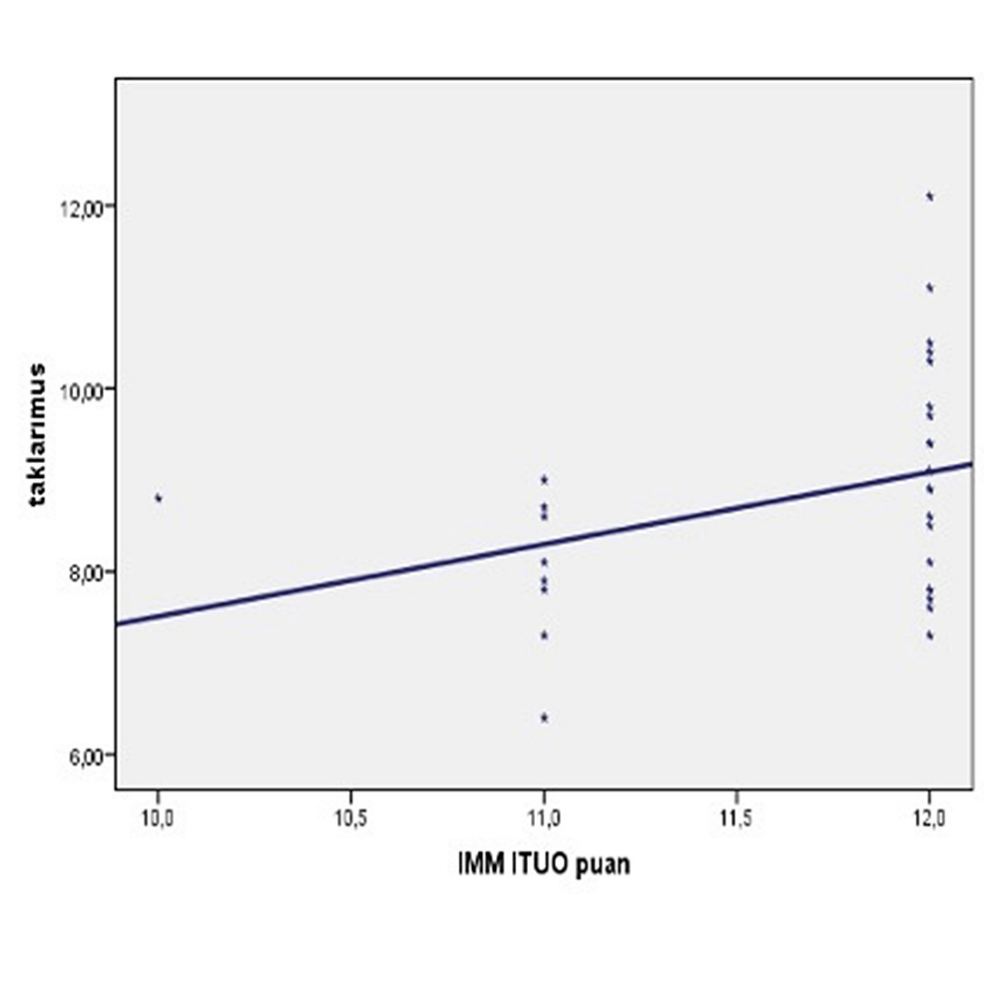
Correlation of tacrolimus levels and ITAS score ITAS - Immunosuppressive Therapy Adherence Scale

In the experimental group, there was a significant positive correlation between tacrolimus levels and ITAS scores (r:0.371; p<0.05). In the control group, there was a significant correlation between tacrolimus levels and ITAS scores (p<0.05). Finally, there was a significant positive correlation in the whole cohort between tacrolimus levels and ITAS scores (r:0.261; p<0.05) (Table 7, Figure 1).

**Table 7:** Correlation between tacrolimus levels and ITAS scores r: Spearman's correlation coefficient *p<0,05 ITAS - Immunosuppressive Therapy Adherence Scale

		ITAS and tacrolimus	
		R	p-value
Experimental (n=30)		0.371	0.044*
Control (n=30)		0.103	0.050
Total (n=60)		0.261	0.044*

## Discussion

Pretest-posttest findings for the experimental group

In organ transplantation, information regarding the drug, rejection symptoms, and how to prevent the negative outcomes associated with life-long immunosuppressive drug therapy can be provided by patient training. The main purpose of this post-transplant training is to help patients cope with health problems and successfully practice self-management behaviors. Patient training is a major responsibility for nurses, playing a major role in creating self-efficacy and transforming patients from dependent individuals into independent and self-sufficient people. This drug training should start from the pre-transplant period and continue in the long term postoperatively. The training aims to eliminate patients' lack of knowledge, correct their mistakes, help them adapt to the post-disease process, and help them comprehend the key points [22-24]. Moors-Tielen et al. (2008) report that 40% of kidney transplant patients stated that forgetting to take their immunosuppressive drugs would not cause serious problems. These patients believed they would be protected against rejection because they took these drugs repeatedly for many years [25]. 

The literature states that training for transplant patients contributes to increased knowledge. Dejean et al. (2004) evaluated drug compliance with a training program and found an increased level of knowledge in the trained group [26]. Similarly, Shemesh et al. (2008) report that their transplant patients had a higher level of knowledge after training for increasing adaptation [27]. The findings obtained here were compatible with the literature. Mean knowledge level scores in the experimental group were 12.17±3.39 before training and 20.73±1.57 after training. This increased level of knowledge after the training was found to be statistically significant (p=0.001; p<0.01) (Table 4). The findings confirm the H2 hypothesis (the experimental group will have a higher level of knowledge following training after kidney transplantation).

ITAS findings

Immunosuppressive drug non-compliance is a common but preventable problem in adult kidney transplants [3]. A systematic review by Jack Lee Low et al. (2014) found that drug compliance rates increased significantly when multidimensional adaptation attempts were performed. However, one-off feedback from nurses and financial aid programs showed little improvement [28]. Studies indicate that practices targeting behavioral risk factors or a combination of behavioral, educational, and effective practices efficiently increase drug compliance [9,11,29]. While multidimensional adaptation interventions are believed to increase the financial burden, the situation is balanced by reducing the health expenses that may occur due to future problems due to non-compliance with drugs. These findings highlight the significance of practices aimed at improving drug compliance as a possible way to improve the clinical outcomes of kidney transplantation. Using various adaptation-enhancing practices, researchers expect to reduce drug non-compliance, which can lead to graft failure, based on the current literature.

Mean total ITAS scores were 11.67±0.55 in the experimental group and 10.70±0.99 in the control group, with a statistically significant difference (p=0.001; p<0.01). It was found that the experimental group had a higher mean total ITAS score than the control group (Table 5). This finding shows that the training increased drug compliance, confirming our H1 hypothesis (after training and interviews, the experimental group will have higher drug compliance than the control group). Our findings were found to be similar to the literature. De Geest (2006) made home visits (educational, behavioral, and social support) and three phone calls to the experimental group for nine months (three-month intervention, six-month follow-up), while the control group only received formal training. Consequently, there was a significant decrease in non-adherence [29]. Cukor et al. (2017) conducted a two-session cognitive-behavioral adherence to the experimental group after surgery to increase drug adherence, and their multivariate analysis indicated a higher risk of non-adherence in the control group [30].

Tacrolimus blood levels and their comparison with ITAS

Some studies evaluating compliance used direct and indirect methods together to increase the reliability of the results, while others used them separately. Here, based on the tacrolimus levels of the experimental group, drug compliance was higher than the control group, with a statistically significant difference consistent with the literature (p<0.05) (Table 6). Foster et al. (2018) evaluated immunosuppressive drug adherence in transplant patients and performed comprehensive psycho-educational to the experimental group. They determined adherence by interviewing the patient's electronic monitoring and considering their tacrolimus levels. Adherence was found to be higher in the experimental group in both methods [24]. Henriksson et al. (2016) analyzed drug compliance using electronic reminders in transplant patients. They evaluated them for one year for outpatient examinations, emergency hospitalizations, kidney biopsies, rejection episodes, kidney functions, and blood concentrations of drugs. The biopsy-confirmed rejection was three times more common in the control group. Also, the mean P-creatinine level was slightly lower in the experimental group than in the control group, while mean tacrolimus levels were similar between the two groups [15]. Reese et al. (2017) evaluated the outcomes of adaptation attempts in three different groups. They examined drug blood levels (twice a week in the first month after transplantation, once a week in the second month after transplantation, and once every two weeks between the third and sixth months). Drug compliance was evaluated at the end of 90 days. Mean compliance rates were 78%, 88%, and 55% [16]. There was no significant difference between the groups regarding mean tacrolimus levels. Considering the tacrolimus levels of the experimental group, drug compliance was higher than the control group, with a statistically significant difference (p<0.05).

In our research, there was a significant positive correlation between tacrolimus levels and ITAS scores in both the experimental and control groups (r:0.261; p<0.05) (Table 7; Figure 1). Based on the similarity of results in both evaluation methods, it is safe to say that using either method alone is sufficient for evaluating drug compliance. Our findings seemingly confirm our H3 (tacrolimus blood levels will be at a level of agreement between the experimental group and the control group) and H4 hypotheses (tacrolimus blood level measurements and using the ITAS scale will have similar outcomes).

Limitations

The sample group of the study consisted of only patients with a kidney transplant in a private hospital. The results of the study could not be generalized to all patients.

## Conclusions

We conclude that performing training and interviews for kidney transplant patients increased their immunosuppressive drug compliance. Tacrolimus blood level measurements, a direct method to evaluate immunosuppressive drug compliance, and using the ITAS scale, an indirect method, proved similar outcomes, suggesting that both evaluation methods are safe.

For improving non-compliance with immunosuppressive drugs, a preventable problem in kidney transplant centers, we suggest implementing practices to increase compliance, ensuring they become widespread, gain continuity, and be added to hospital protocols, ensuring active participation of nurses in this context, developing the current methods for evaluating drug compliance, and conducting further multi-center studies with all types of transplants and larger sample groups including adolescents.
